# Regulation of ABC Drug Efflux Transporters in Human T-Cells Exposed to an HIV Pseudotype

**DOI:** 10.3389/fphar.2021.711999

**Published:** 2021-08-04

**Authors:** Sana-Kay Whyte-Allman, Rupert Kaul, Reina Bendayan

**Affiliations:** ^1^ Department of Pharmaceutical Sciences, Leslie Dan Faculty of Pharmacy, University of Toronto, Toronto, ON, Canada; ^2^ Department of Immunology, Faculty of Medicine, University of Toronto, Toronto, ON, Canada

**Keywords:** ATP-binding cassette transporters, human immunodeficiency virus, mammalian target of rapamycin, proinflammatory cytokines, T-lymphocytes

## Abstract

ATP-binding cassette (ABC) drug efflux transporters could contribute to low intracellular concentrations of antiretroviral drugs in HIV-1 cell reservoirs and sanctuary sites. Furthermore, the functional expression of these transporters could be induced in activated T-cells. Therefore, we investigated the expression of ABC drug efflux transporters in human T-cells exposed to an HIV pseudotype virus (pHIV_NL4-3_), and further examined the potential involvement of the mammalian target of rapamycin (mTOR) signaling pathway in regulating their expression following exposure to pHIV_NL4-3_. Additionally, we investigated the contribution of the drug efflux transporters to the inflammatory response following pHIV_NL4-3_-induced T-cell activation. Human peripheral blood mononuclear cells (PBMCs) were exposed to HIV-1 envelope glycoprotein gp120_IIIB_, pHIV_NL4-3_ and/or mTOR inhibitors. The expression of ABC transporters, T-cell activation marker CD69, mTOR and pHIV_NL4-3_ was assessed in CD4^+^ T-cells by Flow cytometry. mRNA and protein levels of proinflammatory cytokines (IL6, TNFα and INFγ) were examined in PBMCs by qPCR and ELISA analyses, respectively, following exposure to pHIV_NL4-3_ with or without inhibitors of mTOR or ABC transporters. The expression of ABC transporters (P-glycoprotein, breast cancer resistance protein and multi-drug resistance associated protein-1) was significantly increased in CD4^+^ T-cells exposed to pHIV_NL4-3_. Treatment with mTOR inhibitors attenuated pHIV_NL4-3_-induced transporter expression, as well as mRNA and protein levels of IL6, TNFα and INFγ. Additionally, inhibition of P-gp or MRP1 activity resulted in lower concentrations of proinflammatory cytokines in supernatants of PBMC exposed to pHIV_NL4-3_. Herein we present novel data demonstrating that upregulation of ABC drug efflux transporters could involve the mTOR signaling pathway in CD4^+^ T-cells exposed to an HIV pseudotype. These transporters could limit antiretroviral drug penetration in HIV target T-cells. Furthermore, ABC transporters could potentially contribute to HIV-associated proinflammatory cytokine secretion.

## Introduction

A major hallmark of HIV infection and disease progression is the increased activation of both the adaptive and innate immune systems (Meier and Altfeld, 2007; Maartens et al., 2014). Indeed, there is evidence of residual inflammation and increased immune activation in most people living with HIV despite effective antiretroviral therapy (ART) (Maartens et al., 2014). One of the main driving forces for immune activation during infection is the interaction of HIV with the Toll-like receptors expressed on plasmacytoid dendritic cells, leading to the production of proinflammatory cytokines (Maartens et al., 2014). In addition, our group and others have demonstrated that interactions of the HIV envelope glycoprotein (gp120) with the chemokine receptors CXCR4 and CCR5 on cells such as brain macrophages and astrocytes, can induce the secretion of several proinflammatory cytokines such as TNF-α, IL-1β, and IL-6 (Koller et al., 2002; Lee et al., 2003; Ronaldson and Bendayan, 2006; Ronaldson et al., 2010; Ashraf et al., 2014a; Omeragic et al., 2017). Persistent immune activation then facilitates viral infection of activated CD4^+^ T-cells, the preferential targets for HIV-1 production, and may lead to the persistence of HIV within tissue reservoirs (Meier and Altfeld, 2007; Biancotto et al., 2008; Catalfamo et al., 2011).

The expression of several ATP-Binding Cassette (ABC) drug efflux transporters, including P-glycoprotein (P-gp), breast cancer resistance protein (BCRP), and the multidrug resistance-associated protein-1 (MRP1), has been reported to correlate with T-cell activation (Zhang et al., 2006; Ebert et al., 2016). Particularly, Ebert et al., reported that increased expression of P-gp, MRP1 and BCRP correlated with increased expression of the T-cell activation marker CD69 in cells obtained from patients with myeloproliferative neoplasms (Ebert et al., 2016). Additionally, Gupta et al. demonstrated that activation of human T-cells with phytohemagglutinin (PHA), a selective T-cell mitogen, led to an amplification of P-gp expression at the mRNA and protein levels in these cells (Gupta et al., 1992). Furthermore, Liptrott et al. demonstrated increased expression of *ABCB1*/P-gp, *ABCC1*/MRP1, and *ABCC2*/MRP2 mRNA and proteins, as well as reduced accumulation of digoxin and saquinavir, established substrates of ABC transporters, in peripheral blood mononuclear cells (PBMCs) in response to treatment with cytokines such as IL-2 and IFN-γ (Liptrott et al., 2008). On the other hand, studies have also demonstrated that P-gp and MRP1 could participate in the secretion of cytokines in activated PBMCs (Drach et al., 1996; Pawlik et al., 2005; Zhang et al., 2006). Inhibition of P-gp by verapamil (a known P-gp substrate and inhibitor) or anti-P-gp antibodies decreased the secretion of IL-2, IL-4, TNF-a and IFN-y in PHA activated lymphocytes (Drach et al., 1996; Pawlik et al., 2005). MRP1 is also known to be involved in the secretion of several cytokines and leukotrienes (van de Ven et al., 2009), as well as exports glutathione and glutathione disulfide (Ronaldson and Bendayan, 2008). In the context of HIV infection, studies investigating the expression and activity of ABC drug efflux transporters in leukocytes are very limited. HIV-1 infection has been shown to induce the expression and activity of P-gp in the efflux of zidovudine and daunorubicin in the H9 T-cell line and the U937 monocytic cell line (Gupta and Gollapudi, 1993). In addition, Zhang et al. demonstrated higher expression of BCRP in CD4^+^ and CD8^+^ T-cells obtained from people living with HIV-1 (both ART-treated and untreated) compared to healthy donors (Zhang et al., 2014). This implies that activated CD4^+^ T-cells, the preferential target for HIV infection and replication, may also express higher levels of transporters responsible for the efflux of many medications, including antiretroviral agents.

The molecular pathways governing the functional expression of ABC drug efflux transporters in activated T-cells is not clearly understood, but studies have indicated that the mammalian target of rapamycin (mTOR) pathway (Supplementary Figure S1) could be involved (Kuo et al., 2011; Wang et al., 2013; Chen et al., 2015). mTOR is a serine/threonine protein kinase which is central to metabolic control in eukaryotic cells. mTOR exists as two structurally distinct complexes in cells, mTOR complex 1 (mTORC1) and mTORC2, that mediate separate but overlapping cellular functions. mTOR also plays a role in regulating immune responses in T-cells and antigen-presenting cells. Moreover, mTOR activation, as a result of co-stimulatory signals and cytokine production, leads to full T-cell activation, characterized by the elevation of Ca^2+^ and CD69 (Delgoffe and Powell, 2009; Deng et al., 2016). The involvement of mTOR in regulating P-gp expression has been demonstrated in the context of cancer. Wang et al. demonstrated that siRNA mediated knock-down of mTOR resulted in the downregulation of P-gp expression in hepatocellular carcinoma cells (Wang et al., 2013). In addition, Chen et al. demonstrated that selective inhibition of mTOR by compound OSI-027 attenuated doxorubicin-induced overexpression of P-gp in hepatocellular carcinoma cells (Chen et al., 2015). Furthermore, Scherbakova et al. demonstrated that the mTORC1 inhibitor rapamycin downregulated the protein expression of P-gp, BCRP and MRP1 in several human tumor cell lines (Scherbakova et al., 2009). Together, these studies provide evidence that activation of the mTOR signaling pathway could potentially result in increased expression and function of ABC transporters in T-cells.

HIV-1 requires a constant supply of proteins, nucleotides, and energy in order to replicate and generate new virions. Therefore, it has been proposed that this virus may regulate pathways involved in the synthesis of biomolecules, such as mTOR (Kumar et al., 2017). HIV-1 infection can activate the mTOR signaling cascade through the T-cell receptor after the binding of viral gp120 to the CD4 receptor, as well as through a mechanism mediated by viral transactivator of transcription (Tat) (Deng et al., 2016; Kumar et al., 2017). Therefore HIV-induced activation of the mTOR signaling pathway could potentially result in an increased functional expression of ABC transporters in HIV-1 infected T-cells, and in turn this could then contribute to suboptimal antiretroviral drug (ARV) drug penetration within these infected T-cells (Janneh et al., 2005, 2007). Furthermore, these transporters have been implicated in the secretion of proinflammatory cytokines (Drach et al., 1996; Frank et al., 2001; Zhang et al., 2006) and could potentially contribute more broadly to the HIV-associated inflammatory response. In this study, we investigated the role of mTOR in regulating ABC drug efflux transporters in activated T-cells following exposure to HIV-gp120 or HIV_NL4-3_ pseudotyped with the vesicular stomatitis virus glycoprotein (VSVG), as well as assessed the potential role of these transporters in mediating inflammatory cytokine secretion. While, the HIV_NL4-3_ provirus (pNL4-3 GagzipGFP) lacks viral enzymes that are necessary to facilitate its replication cycle, thereby rendering it non-infectious, we anticipate that viral proteins such as Tat could impact the expression of the transporters in PBMCs or T-cells (Hayashi et al., 2005, 2006; Zhou et al., 2016). This study could identify potential mechanisms contributing to low drug penetration in HIV cell reservoirs and sanctuary sites, and persistent immune activation and/or inflammation.

## Materials and Methods

### Ethics Statement

PBMCs were obtained from the blood of symptom-free donors in accordance with the research protocol (REB-12-378) approved by the Unity Health Toronto Research Ethics Board.

### Virus Preparation and Infectivity Assays

The HIV proviral plasmid pNL4-3 GagzipGFP, as well as the pPAX2 packaging construct and VSVG expression vector were kindly provided by Dr. Alan Cochrane (Department of Molecular Genetics, University of Toronto, Toronto, ON, Canada). pNL4-3 GagzipGFP was generated by replacement of the nucleocapsid (NC), protease (PR) and reverse transcriptase (RT) coding regions of pNL4-3 with a leucine zipper sequence fused to enhanced green fluorescence protein (GFP) (Platt et al., 2015). Human embryonic kidney 293T (HEK293T) cells (2.5 × 10^5^) were transfected with pNL4-3, pPAX2 packaging construct and VSVG expression vector using the polyethylenimine transfection protocol to generate HIV_NL4-3_-VSVG pseudotyped virus (pHIV_NL4-3_). Viral supernatant was harvested 72 h post-transfection and stored at −80°C. To assess the infectivity of the virus, 0.5 × 10^6^ HEK293T or 2 × 10^6^ PBMCs were inoculated with serial dilutions (200–1,000 µl) of the viral stock. Infection was initiated through spinoculation at 1,200 × g for 2 h (O’Doherty et al., 2000). Cells were collected and fixed 72 h after infection, and the expression of the enhanced green fluorescence protein (GFP) in cells was determined by flow cytometry. Cells carrying GFP-expressing virus was quantified using FlowJo v10 software. Cells were efficiently transduced with the virus resulting in approximately 90% of HEK293T cells, and up to 60% of PBMCs expressing GFP with the highest volume of viral stock (Supplementary Figure S2). The viral titer was calculated and a multiplicity of infection (MOI) of 0.1 was used in subsequent experiments.

### PBMC Stimulation and Treatment With gp120, pHIV_NL4-3_ and/or Pharmacological Inhibitors

Recombinant HIV envelope glycoprotein 120 (gp120_IIIB_) was obtained from the NIH AIDS Reagent Program (Germantown, MD, United States). gp120_IIIB_ is derived from an X4-tropic viral strain which has been shown to efficiently activate CD4^+^ T-cells, *in vitro* (Doms and Moore, 2010; Islam et al., 2013). Studies have also demonstrated a strong preference of X4-tropic HIV strains to target T-cells *in vitro* compared to other PBMC subsets (Grivel et al., 2000; Woodham et al., 2016), thereby increasing the proportion of infected T-cells. PBMCs isolated from the blood of symptom-free donors were stored in liquid nitrogen vapour phase. These cells were thawed at 37°C and exposed to PHA, or PHA in combination with gp120_IIIB_. Initially, cells were treated with 1–10 µg/ml PHA or 0.25–1 µg/ml gp120-_IIIB_ for 24–72 h as previously described (Pawlik et al., 2005; Zimmermann et al., 2015). The concentrations of gp120_IIIB_ (1 µg/ml) and PHA (2.5 µg/ml) that yielded consistent results were then used for subsequent experiments. In addition, simultaneous changes in the expression of the three transporters following exposure to these agents was observed at 48 h, as such, cells were collected for flow cytometry analyses after a 48 h treatment duration. For experiments with pHIV_NL4-3_, PBMCs were cultured in IL-2 media (complete RPMI containing 10 U/ml IL-2) to induce cell proliferation. Following 72 h PHA stimulation, cells were exposed to pHIV_NL4-3_ at an MOI of 0.1 in the presence of the infection reagent polybrene (8 µg/ml); unexposed control cells contained polybrene without the virus. A heat-inactivated pHIV_NL4-3_ control, obtained by heating at 57°C for 45 min, was used as an additional negative control to demonstrate specificity of the viral response (Supplementary Figure S3). Infection was initiated through spinoculation at 1,200 × g for 2 h. Cells were then incubated at 37°C for 48 h to determine the effects of the virus on T-cell activation and transporter expression by flow cytometry. To investigate the involvement of the mTOR pathway, cells were treated with mTORC1 inhibitor rapamycin (5 µM), mTORC2 inhibitor JR-AB2-011 (50 µM), dual mTOR inhibitor OSI-027 (25 µM) which inhibits both mTORC1 and mTORC2 (Bhagwat et al., 2011), or vehicle control (DMSO) for 48 h following spinoculation. Initial dose-response experiments were performed for each inhibitor to determine an appropriate concentration to use in the subsequent experiments. To assess the function of the transporters in proinflammatory cytokine export, cells were exposed to P-gp inhibitors verapamil or PSC833, MRP1 inhibitor MK571 or vehicle for 48 h following spinoculation with the virus. The cells were centrifuged prior to flow cytometry analyses, and supernatants were collected and stored at –80°C for enzyme linked immunoabsorbant assay (ELISA).

### Flow Cytometry

PBMCs were collected by centrifugation 48 h following exposure to pHIV_NL4-3_ and/or drug treatment and stained extra-cellularly with fluorochrome-conjugated antibodies to detect surface receptors CD3, CD4 and CD69, in the presence of the Zombie aqua cell viability dye (Supplementary Table S1) to exclude dead cells. Cells were then fixed and permeabilized prior to intracellular staining of ABC transporters P-gp, BCRP and MRP1 and phosphorylated pmTOR (aa 2448) using the True Nuclear Transcription Factor permeabilization kit (BioLegend, San Diego, CA, United States) and manufacturer’s protocol. Cells were acquired on a Beckman Coulter Cytoflex-S benchtop cytometer to assess the expression of CD69, ABC transporters, pmTOR and GFP in CD3^+^CD4^+^ T-cells. Fluorescence minus one (FMO) strategy was used to set the gates for positive events, as previously demonstrated (Whyte-Allman et al., 2020). Data analysis was performed using FlowJo v10 software and GraphPad prism 7.03. See Supplementary Table S1 for a list of fluorochrome-conjugated antibodies and suppliers used in these experiments.

### Enzyme Linked Immunoabsorbant Assay

Single-analyte colorimetric sandwich ELISA kits (QIAGEN, Toronto, ON, Canada) for human IL-6, INFγ and TNF-α were used to detect the secretion of these cytokines in cell culture supernatants of PBMCs exposed to pHIV_NL4-3_ in the presence or absence of a vehicle (DMSO) control or one of the following inhibitors: the mTOR inhibitor OSI-027 (25 µM), P-gp inhibitors verapamil (10 or 50 µM) or PSC833 (5 µM), or the MRP1 inhibitor MK571 (10, 50, or 100 µM). These are HIV-inducible cytokines known to be produced by T-cells, and have been shown to be transported by the efflux transporters in previous studies (Kedzierska and Crowe, 2001; Pawlik et al., 2005; Zhang et al., 2006; Freeman et al., 2016). The concentrations of these compounds were chosen based on previous studies by our group and others to inhibit their transport function in PBMCs without exerting toxic effects (Drach et al., 1996; Pawlik et al., 2005; Zhang et al., 2006; Whyte-Allman et al., 2020). The assays were performed according to the manufacturer’s instructions. Standard curves were generated using appropriate antigen standards and absorbance was read at 450 nm and converted to pg/ml.

### Real-Time Quantitative Polymerase Chain Reaction

Real-time quantitative Polymerase Chain Reaction (qPCR) was applied to determine the transcript levels of inflammatory cytokines as previously described by our group (Whyte-Allman et al., 2017; Omeragic et al., 2019, 2020). Briefly, total RNA was extracted from cells using TRizol reagent. The concentration of RNA was quantified spectrophotometrically by measuring absorbance at 260 nm. Extracted RNA (2000 ng) was treated with amplification grade DNase I (0.1 U/ml) to remove contaminating genomic DNA. The high-capacity cDNA reverse transcriptase kit (Applied Biosystems, Waltham, MA, United States) was used to synthesize first-strand cDNA. Human primers using TaqMan technology were purchased from Life Technologies (Burlington, ON, Canada) for the following genes: TNFα (Hs00174128_m1), IL6 (Hs00174131_m1) and IFNγ (Hs00989291_m1) and housekeeping gene glyceraldehyde 3-phosphate dehydrogenase (GAPDH; Hs02758991_g1). Expression levels were normalized to the housekeeping gene, GAPDH and compared to vehicle-treated control group using the comparative C_T_ (ΔΔC_T_) method and expressed as fold expression (2^−ΔΔCT^) to assess the relative difference in mRNA expression for each gene. GAPDH levels in the PBMCs remained consistent between experiments.

### Statistical Analysis

All experiments were performed in cells isolated from the blood of at least three adult human donors. Results are expressed as the mean ± SEM. Comparison between groups was performed as appropriate by applying one-way ANOVA with Bonferroni’s multiple comparisons test using GraphPad Prism 7.03 software (GraphPad Software Inc., San Diego, CA, United States); *p* < 0.05 was considered statistically significant.

## Results

### HIV-gp120 Enhances T-Cell Activation and ABC Transporter Expression

**FIGURE 1:**
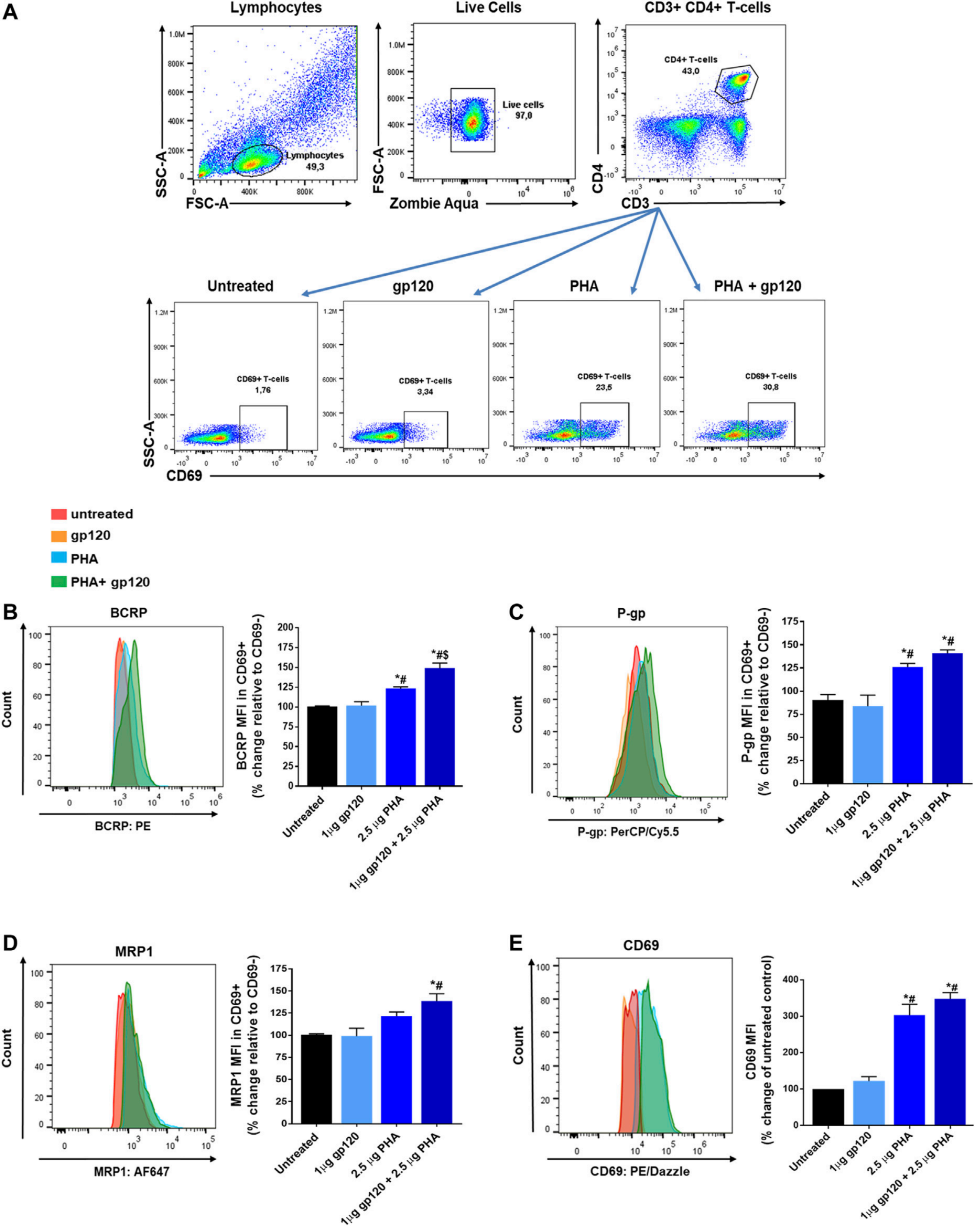
Expression of BCRP, P-gp, MRP1 and CD69 in PHA and/or gp120 activated CD4+ T-cells. **(A)** Representative gating strategy for CD4^+^ T-cells isolated from PBMCs; panels from left to right show lymphocytes selected based on light scattering properties, viable (negative for Zombie Aqua) single cells were then selected for CD3^+^CD4^+^ expression, CD69 expression was determined in untreated, gp120 treated, PHA treated, or gp120 + PHA treated CD4^+^ T-cells. **(B**–**D)** Mean percent change ± SEM of the expression (median fluorescence intensity, MFI) of each ABC transporter in CD4^+^CD69^+^ T-cells relative to their expression in non-activated CD4^+^CD69^−^ T-cells. **(E)** Mean percent change ± SEM in the expression of CD69 in gp120 and/or PHA treated cells compared to untreated cells. Left panels show representative histograms corresponding to bar charts on the right **(B**–**E)**. Statistical analyses were performed using one-way ANOVA with Bonferroni’s multiple comparisons test, *p* < 0.05. *, statistically significant difference compared to untreated control cells; *#*, statistically significant difference compared to cells treated with gp120 only; *$*, statistically significant difference compared to cells treated with PHA only, *n* = 3 donors.

To investigate whether the viral envelope protein gp120 could enhance T-cell activation and transporter expression, PBMCs were exposed to PHA, gp120_IIIB_ or PHA in combination with gp120_IIIB_. The expression of the ABC transporters or the T-cell activation marker CD69 in CD4 T-cells was then quantified and compared among these treatment groups following flow cytometry analyses. During flow cytometry analyses, cells were examined for viability using the zombie aqua cell viability dye and gated based on their expression of the CD3, CD4, and CD69 T-cell surface markers (Figure 1A). T-cell activation was determined by the expression of the CD69 T-cell activation marker. The expression of P-gp, BCRP and MRP1 was then evaluated in CD3^+^CD4^+^CD69^+^ T-cells and normalized to their expression in CD3^+^CD4^+^CD69^−^ T-cells. As expected, PHA treatment increased the surface expression of the T-cell activation marker CD69 (203%) compared to untreated control cells, as well as ABC transporters P-gp (25%), BCRP (23%) and MRP1 (21%; Figures 1B–D). Overall, the highest expression of CD69 (248%), P-gp (41%), BCRP (49%) and MRP1 (39%) was observed in cells that were exposed to PHA + gp120. Although a higher level of expression was observed in cells treated with PHA + gp120 compared to PHA alone, the differences between these two groups did not reach statistical significance, except for BCRP. Exposing unstimulated cells to gp120 alone did not alter the expression of the transporters or CD69, suggesting its preferential interaction with PHA-stimulated T-cells. Together, these results indicate subtle effects of gp120 in enhancing T-cell activation and transporter expression in activated T-cells.

### Induction of ABC Transporters in pHIV_NL4-3_ Activated CD4^+^ T-Cells

**FIGURE 2:**
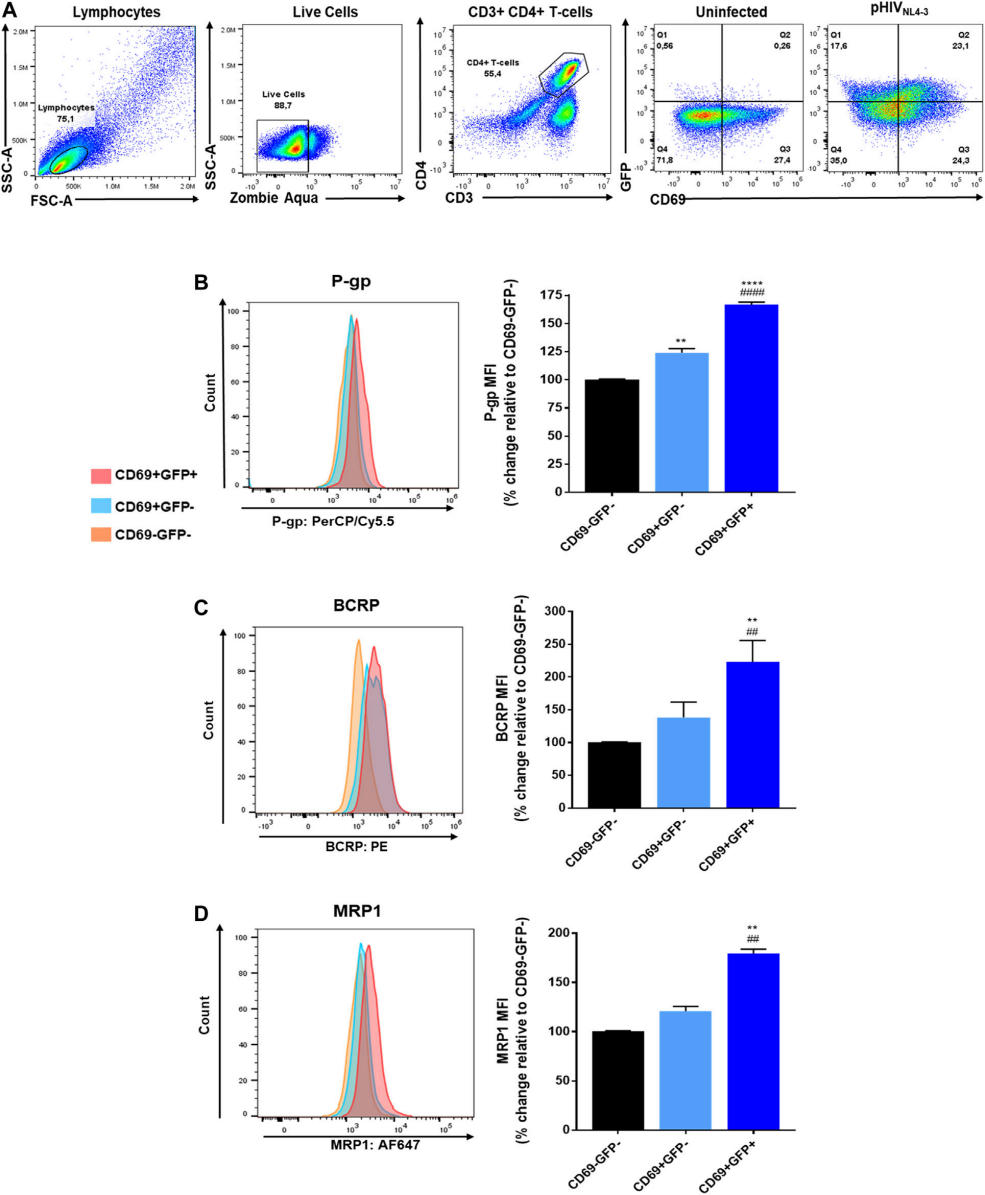
Expression of P-gp, BCRP and MRP1 in pHIV_NL4-3_ activated CD4+ T-cells. pHIV_NL4-3_ in cells is demonstrated by the expression of GFP (GFP+). **(A)** Representative gating strategy for CD4^+^ T-cells isolated from PBMCs exposed to pHIV_NL4-3_ or untreated control; panels from left to right show lymphocytes selected based on light scattering properties, viable (negative for Zombie Aqua) single cells were then selected for CD3^+^CD4^+^ expression, then the expression of CD69 and/or GFP was determined in CD4^+^ T-cells. **(B**–**D)** Results are shown as mean percent change ± SEM in the expression (median fluorescence intensity, MFI) of each ABC transporter in activated cells without the virus (CD69+GFP-), or activated cells carrying pHIV_NL4-3_ (CD69+GFP+), compared to their expression levels in control non-activated (CD69-GFP-) cells. Left panels show representative histograms corresponding to bar charts on the right. Statistical analyses were performed using one-way ANOVA with Bonferroni’s multiple comparisons test. *, statistically significant difference compared to CD69-GFP- cells; #, statistically significant difference compared to CD69+GFP- cells, *n* = 3 donors. **, *p* < 0.01; ****, *p* < 0.0001; ##, *p* < 0.01; ####, *p* < 0.0001.

Next, we investigated the effects of pHIV_NL4-3_ in inducing the expression of the transporters in the T-cells. The expression of P-gp, BCRP and MRP1 was evaluated in PHA-stimulated CD4^+^ T-cells that were exposed to pHIV_NL4-3_ for 48 h and compared to unexposed control cells. Cells expressing GFP were considered pHIV_NL4-3_ exposed. The expression level of P-gp, BCRP and MRP1 was then evaluated and compared in CD69-GFP- (non-activated, non-pHIV_NL4-3_-exposed), CD69+GFP- (activated, non-pHIV_NL4-3_-exposed), or CD69+GFP+ (activated, pHIV_NL4-3_-exposed) CD4^+^ T-cells (Figure 2). The results demonstrated that exposure to pHIV_NL4-3_ (CD69+GFP+) significantly enhanced P-gp, BCRP and MRP1 by 66, 123 and 80%, respectively, when compared to CD69-GFP- cells (Figure 2). Their expression was also significantly higher in CD69+GFP+ compared to CD69+GFP- cells. These results suggest that HIV infection can induce ABC drug efflux transporter expression in CD4^+^ T-cells.

### Role of mTOR in CD4^+^ T-Cell Activation and Induction of ABC Transporters in pHIV_NL4-3_ Exposed CD4^+^ T-Cells

**FIGURE 3:**
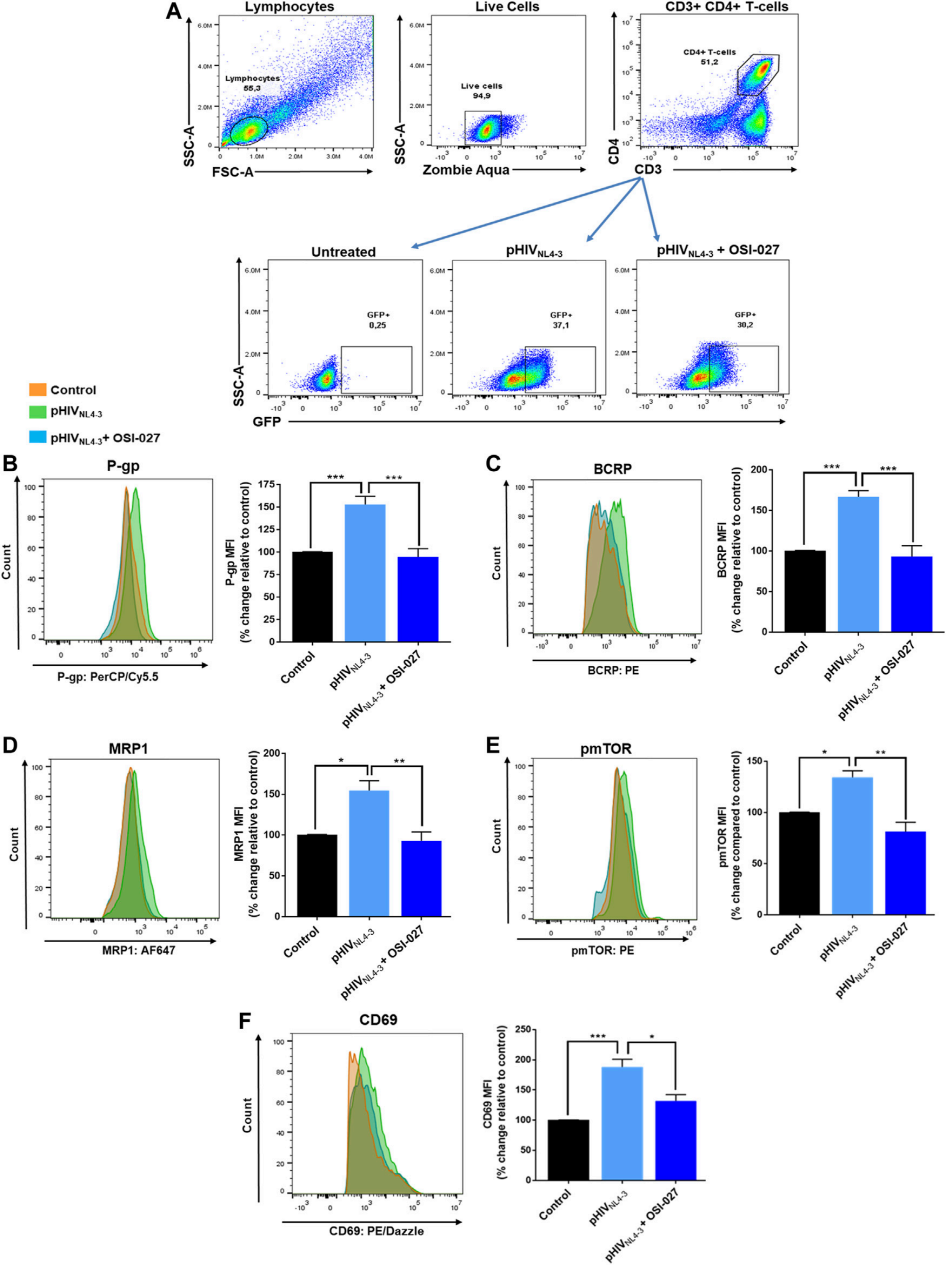
mTOR inhibitor OSI-027 reverses pHIV_NL4-3_ -mediated induction of transporter expression. **(A)** Representative gating strategy for CD4^+^ T-cells isolated from PBMCs; panels from left to right show lymphocytes selected based on light scattering properties, viable (negative for Zombie Aqua) single cells were then selected for CD3^+^CD4^+^ expression. The expression of GFP is shown in untreated, pHIV_NL4-3_ and/or OSI-027 (25 µM) treated CD4^+^ T-cells after 48 h exposure. **(B**–**F)** Representative histograms **(left)** and corresponding bar charts **(right)** are shown. Results are shown as mean percent change ± SEM in the expression (MFI) of P-gp **(B)**, BCRP **(C)**, MRP1 **(D)**, pmTOR **(E)** and CD69 **(F)** in untreated (vehicle control), pHIV_NL4-3_, and/or OSI-027 treated CD4^+^ T-cells. Statistical analyses were performed using one-way ANOVA with Bonferroni’s multiple comparisons test; *p* < 0.05 was considered statistically significant, *n* = 3–4 donors. *, *p* < 0.05; **, *p* < 0.01; ***, *p* < 0.001.

To determine if the mTOR signaling pathway is involved in regulating the expression of ABC transporters in activated and/or pHIV_NL4-3_ exposed T-cells, the dual mTOR inhibitor OSI-027 was used to inhibit the phosphorylation and activity of mTOR (aa 2448) (Bhagwat et al., 2011). Following treatment, the expression of CD69, P-gp, BCRP, MRP1 and phosphorylated mTOR (pmTOR, aa 2448) was evaluated in CD4^+^ T-cells (Figure 3). OSI-027 had no significant effect on the percentage of cells carrying GFP-expressing virus (average frequencies of 32% in pHIV_NL4-3_ group vs. 30% in pHIV_NL4-3_ + OSI-027 group, Figure 3A). Exposure to pHIV_NL4-3_ induced the expression of P-gp (52%), BCRP (66%) and MRP1 (54%), however, treatment with OSI-027 significantly reversed these effects, bringing the expression of these proteins to basal levels (Figures 3B–D). Exposure to pHIV_NL4-3_ also induced activation of mTOR, resulting in increased pmTOR (34%) expression. OSI-027 significantly decreased the levels of pmTOR following treatment (Figure 3E). Lastly, the expression of CD69 was also increased (88%) in pHIV_NL4-3_ exposed T-cells, suggesting enhanced T-cell activation. Inhibition of pmTOR with OSI-027 also reversed the pHIV_NL4-3_-induced CD4^+^ T-cell activation, which was demonstrated by reduced levels of CD69 expression on these cells (Figure 3F). Overall, these results suggest a potential role of mTOR in regulating T-cell activation and expression of ABC drug efflux transporters following exposure to pHIV_NL4-3_.

**FIGURE 4:**
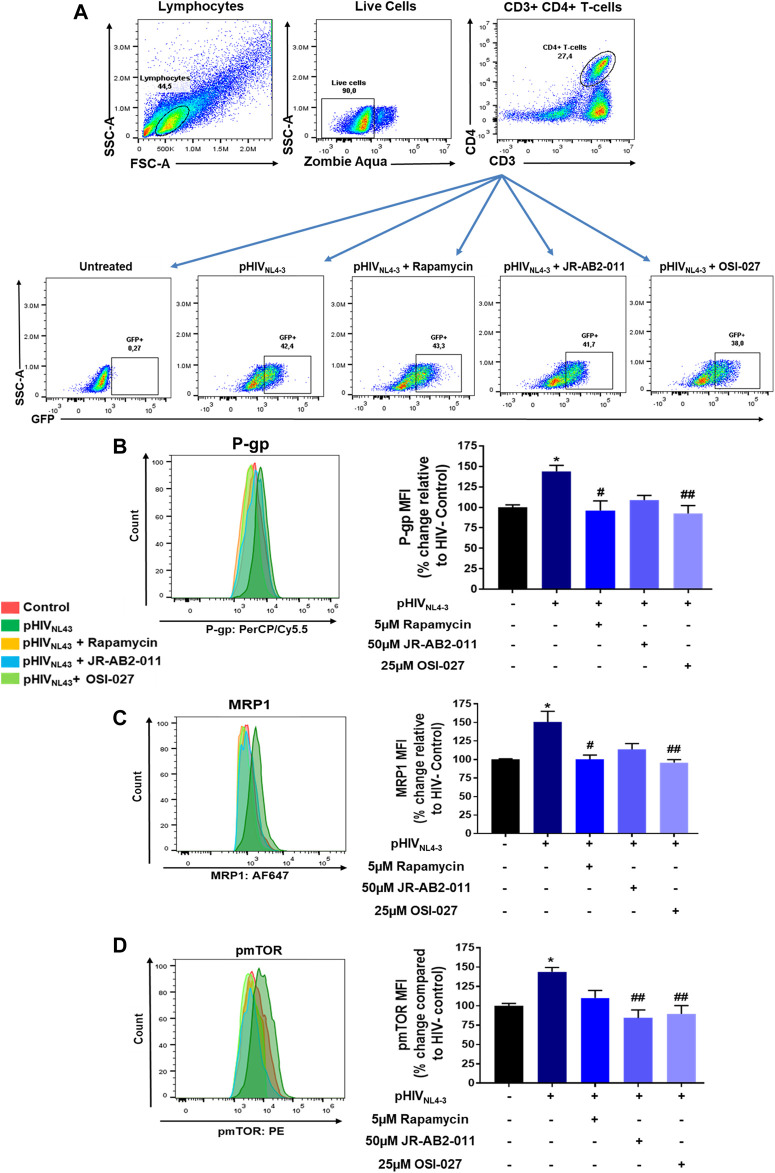
Involvement of mTORC1 and mTORC2 in regulating pHIV_NL4-3_ exposed transporter expression. **(A)** Representative gating strategy for CD4^+^ T-cells isolated from PBMCs; panels from left to right show lymphocytes selected based on light scattering properties, viable (negative for Zombie Aqua) single cells were then selected for CD3^+^CD4^+^ expression. The expression of GFP is shown in untreated, pHIV_NL4-3_, rapamycin (5 µM), JR-AB2-011 (50 µM) and/or OSI-027 (25 µM) treated CD4^+^ T-cells after 48 h incubation time. **(B**–**D)** Representative histograms **(left)** and corresponding bar charts **(right)** are shown. Results are shown as mean percent change ± SEM in the expression (MFI) of P-gp **(B)**, MRP1 **(C)**, pmTOR **(D)** in untreated (vehicle control), pHIV_NL4-3_, rapamycin, JR-AB2-011 and/or OSI-027 treated CD4^+^ T-cells. Multiple comparisons were performed using one-way ANOVA and Bonferroni’s post-hoc analysis; *p* < 0.05 was considered statistically significant, *n* = 3 donors. *, statistically significant difference compared to untreated control; #, statistically significant difference compared to pHIV_NL4-3_ only. *, *p* < 0.05; #, *p* < 0.05; ##, *p* < 0.01.

We further investigated the involvement of each mTOR subunit in the regulation of P-gp and MRP1 in pHIV_NL4-3_ infected CD4^+^ T-cells by selectively inhibiting mTORC1 with rapamycin (Wang et al., 2013) or mTORC2 with JR-AB2-011 (Benavides-Serrato et al., 2017) (Figure 4). Due to sample limitations, we only assessed the expression of P-gp, MRP1 and pmTOR in these experiments. The expression of these transport proteins in CD4^+^ T-cells was determined in each treatment group or control. Rapamycin treatment significantly decreased the expression of pHIV_NL4-3_-induced P-gp and MRP1 by 40 and 43%, respectively, while JR-AB2-011 significantly decreased the expression of pmTOR by 60%. Although, rapamycin resulted in a 33% decrease in mTOR phosphorylation, this did not reach statistical significance. This is likely due to the small sample size. Overall, mTORC1 inhibition compared to mTORC2, was not found significantly different. However, mTOR inhibition with the dual inhibitor OSI-027 demonstrated significant effects in reversing the pHIV_NL4-3_-induced expression of these transporters. More work with a larger sample size is needed to further investigate the involvement of respective mTOR complexes in the regulation of these transporters following HIV infection.

### mTOR Regulates Proinflammatory Cytokine Production/Secretion Following Exposure to pHIV_NL4-3_

**FIGURE 5:**
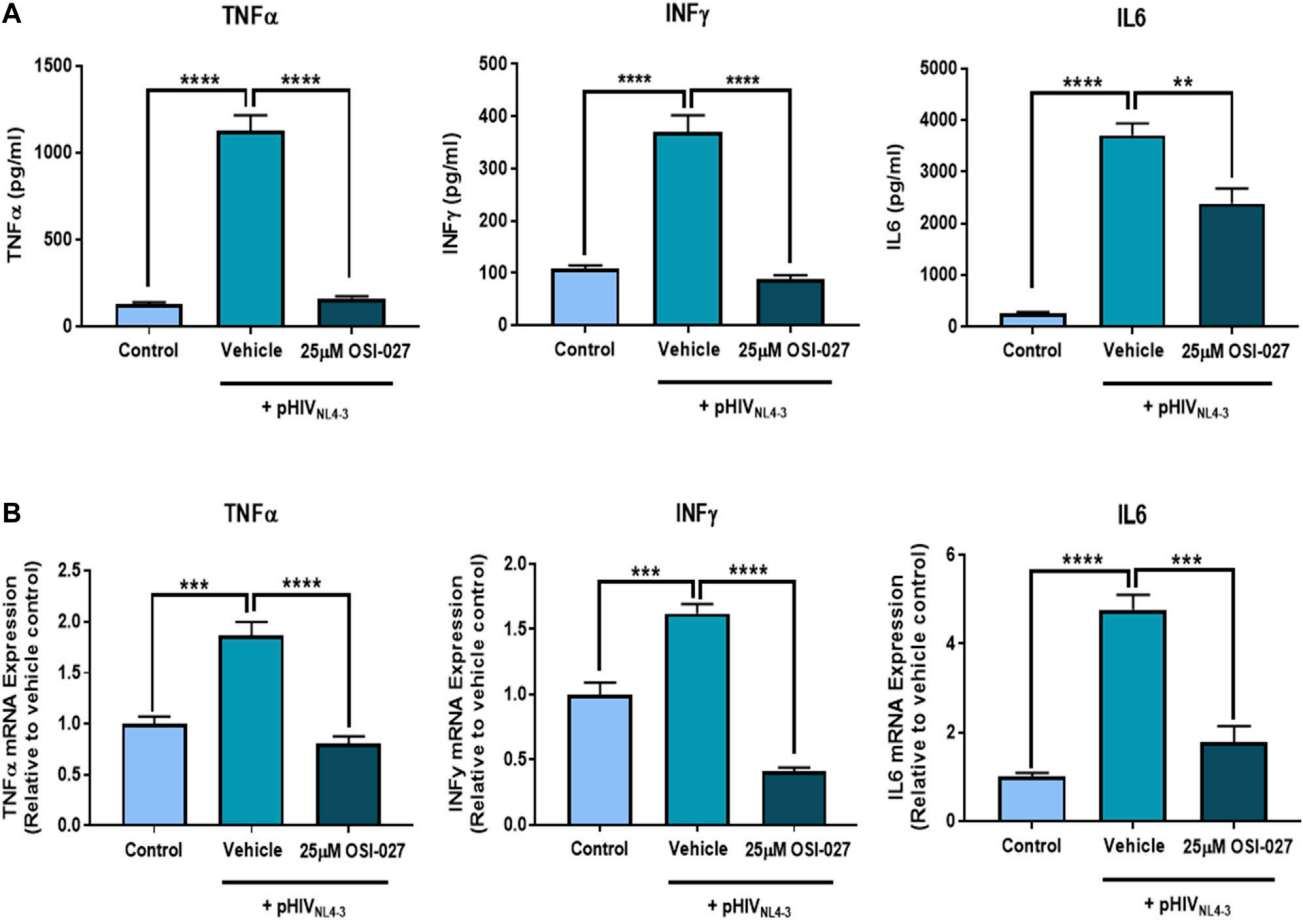
The effects of mTOR inhibitor OSI-027 on the expression and secretion of proinflammatory cytokines from pHIV_NL4-3_ exposed PBMCs. PBMCs and supernatants were collected following exposure to vehicle, pHIV_NL4-3_ only, or pHIV_NL4-3_ in the presence of 25 µM OSI-027 for 48 h. **(A)** cytokine concentrations in cell culture supernatants; results are expressed as mean ± SEM (*n* = 3 donors). **(B)** Cytokine mRNA levels were measured using qPCR. GAPDH was used as the housekeeping gene. Results are expressed as mean ± SD (*n* = 3 donors). Asterisks represent data points significantly different from cells exposed to HIV only. Multiple comparisons were performed using one-way ANOVA and Bonferroni’s post-hoc analysis; *p* < 0.05 was considered statistically significant. **, *p* < 0.01; ***, *p* < 0.001; ****, *p* < 0.0001.

MTOR is known to play a role in modulating immune responses, therefore we evaluated the effects of mTOR inhibition on the production of proinflammatory cytokines following exposure to pHIV_NL4-3_. Exposure of PBMCs to pHIV_NL4-3_ induced a robust inflammatory response which resulted in elevated cellular mRNA levels and secreted protein concentrations of the proinflammatory cytokines TNFα, INFγ and IL6 (Figure 5). Inhibiting the mTOR pathway with OSI-027 for 48 h significantly attenuated the pHIV_NL4-3_-induced mRNA expression and extracellular protein concentrations of TNFα, INFγ and IL6 in PBMCs. These results demonstrate an inflammatory response following exposure to pHIV_NL4-3_ and confirm that mTOR plays a role in regulating proinflammatory cytokine production from activated T-cells as previously reported (Herrero-Sánchez et al., 2016).

### Involvement of P-gp and MRP1 in the Release of Proinflammatory Cytokines Following Exposure to pHIV_NL4-3_

**FIGURE 6:**
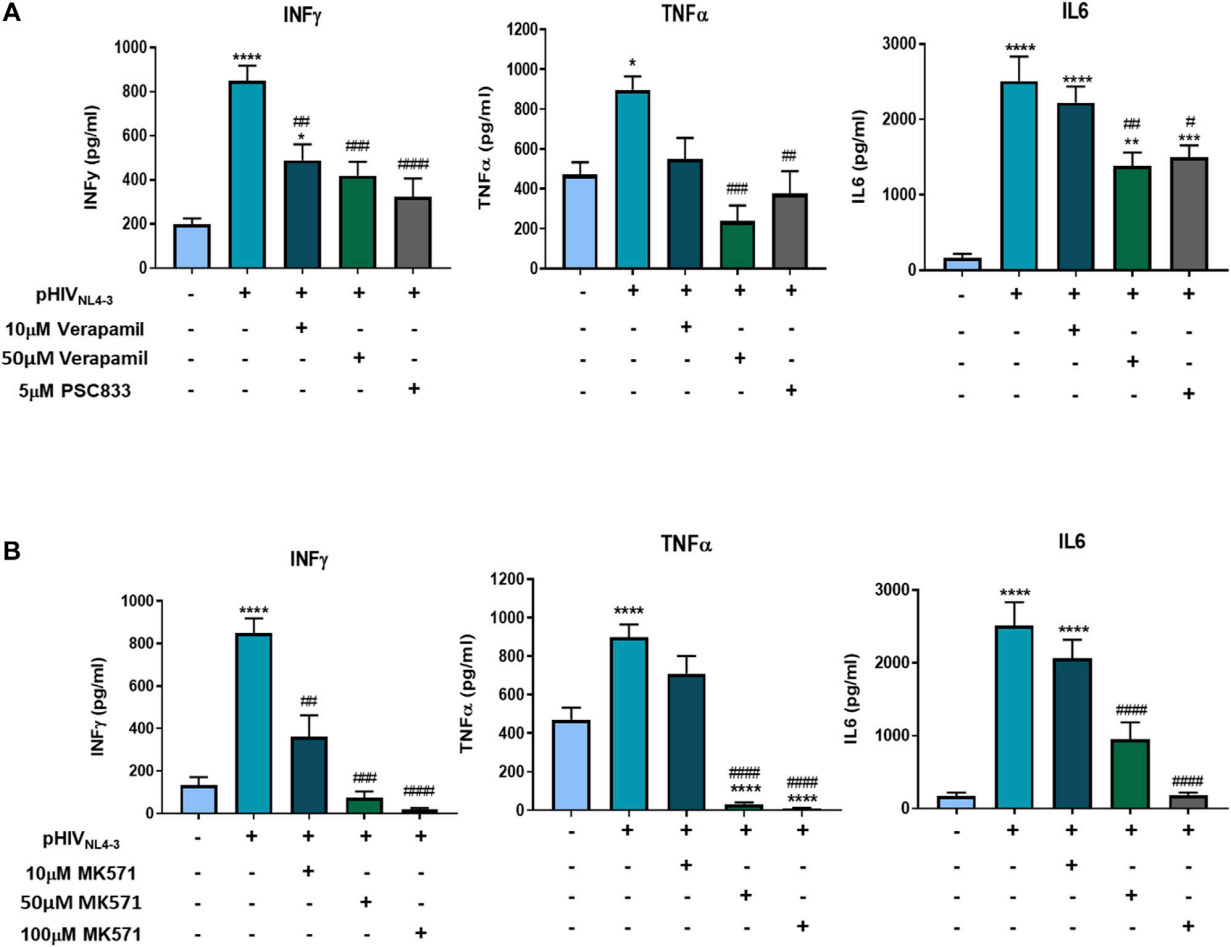
P-gp and MRP1 demonstrate involvement in proinflammatory cytokine secretion following pHIV_NL4-3_ exposure. The effects of P-gp inhibitors verapamil and PSC833 **(A)** or MRP1 inhibitor MK571 **(B)** on the release of proinflammatory cytokines from pHIV_NL4-3_ exposed PBMCs. Supernatants were collected from cells exposed to vehicle, pHIV_NL4-3_ only, or pHIV_NL4-3_ in the presence of inhibitors for 48 h. Results are expressed as mean ± SEM of experiments performed in cells obtained from three donors (*n* = 3 donors). Multiple comparisons were performed using one-way ANOVA and Bonferroni’s post-hoc analysis; *p* < 0.05 was considered statistically significant. *, statistically significant difference compared to untreated control; #, statistically significant difference compared to pHIV_NL4-3_ only. *, *p* < 0.05; **, *p* < 0.01; ***, *p* < 0.001; ****, *p* < 0.0001; #, *p* < 0.05; ##, *p* < 0.01; ###, *p* < 0.001; ####, *p* < 0.0001.

In the context of cancer or autoimmune disease, studies have demonstrated that drug efflux transporters such as P-gp and MRP1 could participate in the secretion of proinflammatory cytokines following activation of T-cells (Drach et al., 1996; Pawlik et al., 2005; Zhang et al., 2006). As these transporters are upregulated during pHIV_NL4-3_-induced T-cell activation, we further investigated their potential roles in the release of proinflammatory cytokines following exposure to the viral pseudotype. To examine the functional involvement of these transporters in the release of proinflammatory cytokines, PBMCs were treated with well-characterized transporter inhibitors following pHIV_NL4-3_ exposure. Specifically, P-gp was inhibited with PSC833 and verapamil, while MK571 was used to inhibit MRP1. The results demonstrated elevated cytokine levels in the supernatants of cells exposed to pHIV_NL4-3_ when compared to uninfected control cells (Figure 6). Treating cells with P-gp or MRP1 inhibitors significantly decreased proinflammatory cytokine concentrations in the supernatants of cells exposed to pHIV_NL4-3_. These data suggest that ABC transporters could potentially contribute to proinflammatory cytokine export from HIV-infected T-cells.

### mRNA Expression of Proinflammatory Cytokines Following Exposure to pHIV_NL4-3_ and/or P-gp and MRP1 Inhibitors

**FIGURE 7:**
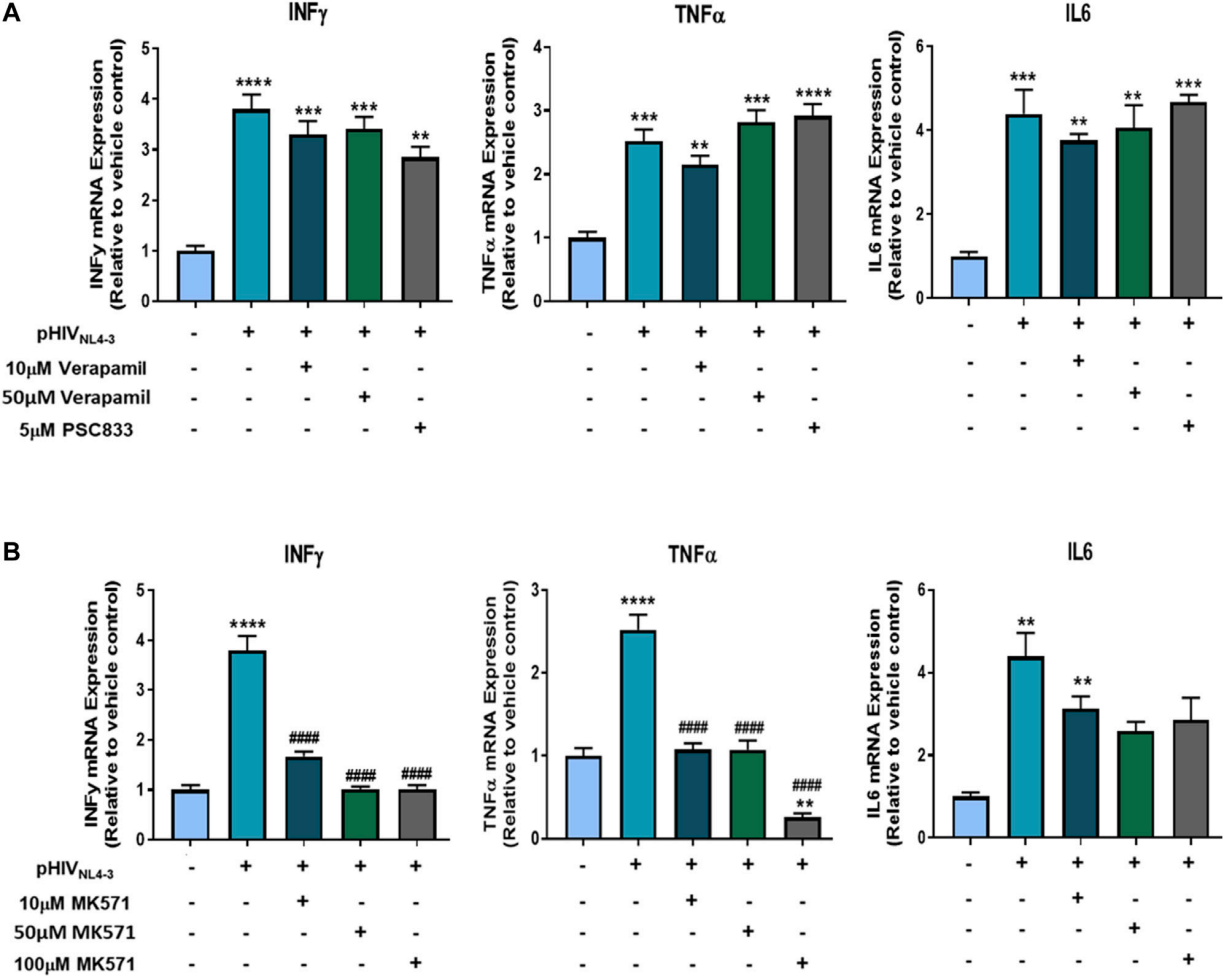
Effects of P-gp and MRP1 inhibitors on proinflammatory cytokine mRNA expression following pHIV_NL4-3_ exposure. The effects of P-gp inhibitors verapamil and PSC833 **(A)** or MRP1 inhibitor MK571 **(B)** on the mRNA expression of proinflammatory cytokines from pHIV_NL4-3_ exposed PBMCs. Cells were exposed to vehicle, pHIV_NL4-3_ only, or pHIV_NL4-3_ in the presence of inhibitors for 48 h. mRNA levels were measured using qPCR. GAPDH was used as the housekeeping gene. Results are expressed as mean ± SD of experiments performed in cells obtained from three donors (*n* = 3 donors). Multiple comparisons were performed using one-way ANOVA and Bonferroni’s post-hoc analysis; *p* < 0.05 was considered statistically significant. *, statistically significant difference compared to untreated control; #, statistically significant difference compared to pHIV_NL4-3_ only. **, *p* < 0.01; ***, *p* < 0.001; ****, *p* < 0.0001; ####, *p* < 0.0001.

We further investigated if inhibiting P-gp or MRP1 affected the production of proinflammatory cytokines, rather than their export, by assessing their expression at the mRNA level following exposure to the transporter inhibitors. The corresponding mRNA expression of these cytokines was assessed in PBMCs exposed to pHIV_NL4-3_ and treated with transporter inhibitors compared to cells exposed to the virus only or negative control (Figure 7). The results demonstrate significant increase in the mRNA expression of IL-6, TNFα, INFγ in cells exposed to pHIV_NL4-3_. P-gp inhibitors verapamil and PSC833 did not alter the pHIV_NL4-3_-induced mRNA expression of these cytokines. However, MRP1 inhibitor MK571 significantly attenuated pHIV_NL4-3_-induced cytokine mRNA expression, suggesting its potential role in contributing to the transcriptional processes of these proinflammatory cytokines in the context of HIV infection. To the best of our knowledge this is the first study investigating the effects of these inhibitors on the mRNA expression of proinflammatory cytokines in the context of HIV infection.

## Discussion

It is well established that activation of CD4^+^ T-cells enables their infection by HIV, and that the percentage of activated T-cells is increased in people with treated HIV infection (Biancotto et al., 2008; Catalfamo et al., 2011). The expression of ABC drug efflux transporters could also be increased in HIV-infected T-cells, however the mechanisms underlying their upregulation in these HIV cellular targets have not been investigated. Upregulation of ABC transporters in T-cells could potentially contribute to persistent infection by reducing ARV penetration in these cells, and possibly influencing HIV-associated inflammation. We previously demonstrated functional activity of these transporters in restricting the cellular permeability of their substrates in human T-cells (Whyte-Allman et al., 2020). However, in this study we investigated their expression and regulation by mTOR during T-cell activation, as well as their functional roles in proinflammatory cytokine secretion, following exposure of PBMCs to HIV-gp120 or pHIV_NL4-3_, an X4-tropic HIV strain pseudotyped with VSVG. Overall, our results demonstrated that pHIV_NL4-3_ induced T-cell activation and inflammatory response, resulting in the upregulation of P-gp, BCRP and MRP1 via the mTOR signaling pathway. These transporters also demonstrated a potential indirect role in the export of proinflammatory cytokines when PBMCs were exposed to pHIV_NL4-3_. Together, the data suggest that ABC transporters could contribute to sustained viral replication and/or reduced ARV intracelluar concentrations in cells known to form HIV reservoirs.

PBMCs were exposed to a concentration of gp120_IIIB_ (i.e., 1 µg/ml) that is at the upper end of the reported plasma concentration range (120–960 ng/ml) for HIV-infected individuals (Cummins et al., 2010). Overall, significant increases in the expression of the efflux transporters P-gp, BCRP and MRP1, as well as the T-cell activation marker CD69, were observed when cells were treated with gp120_IIIB_ + PHA. However, the effects of this single viral protein were very mild, and in most cases did not reach significance, when compared to cells that were treated with PHA only. We then evaluated the effects of the HIV pseudotype, pHIV_NL4-3_, on the expression of the drug efflux transporters P-gp, BCRP, MRP1 and CD69 and observed significant increases in their expression following exposure to the virus. Zhang et al. previously demonstrated that compared to HIV-negative donors, BCRP expression was higher in CD4^+^ and CD8^+^ T-cells obtained from both ART-treated and ART-naïve individuals living with HIV (Zhang et al., 2014). In addition, higher mRNA expression of P-gp, MRP1, MRP4 and MRP5 was demonstrated in PBMCs isolated from individuals with HIV compared to control (Turriziani et al., 2008). Furthermore, a positive correlation between HIV-1 plasma viral load and P-gp activity in primary CD4^+^ and CD4^+^CD45RA-memory T-cells was demonstrated (Minuesa et al., 2016). Our group and others also observed increased expression of ABC drug transporters in memory T-cells when compared to their naïve counterparts (Zhang et al., 2006; Whyte-Allman et al., 2020), further suggesting that these transporters are induced in activated T-cells.

We identified the mTOR signaling pathway as a potential mechanism involved in the upregulation of ABC drug efflux transporters and CD69 in CD4^+^ T-cells exposed to pHIV_NL4-3_. HIV requires a constant supply of proteins, nucleotides and energy to maintain viral replication. Therefore it has been hypothesized that the virus induces pathways such as mTOR, which plays a major role in biosynthesis and metabolism (Kumar et al., 2017). Furthermore, Kumar et al. provided evidence that HIV activates mTOR in Jurkat cells (a latent T-cell model), HeLa cells, and peripheral blood lymphocytes (Kumar et al., 2017). Herein, treatment with the dual mTOR inhibitor OSI-027 attenuated the pHIV_NL4-3_-induced expression of P-gp, BCRP, MRP1, CD69 and pmTOR. These results corroborate previous studies which demonstrated that gene silencing or selective inhibition of mTOR decreased the expression of transporters such as P-gp in the context of cancer (Pop et al., 2009; Wang et al., 2013; Chen et al., 2015). We sought to examine the involvement of each mTOR subunit in regulating P-gp and MRP1 expression in pHIV_NL4-3_ exposed cells, by selectively inhibiting mTORC1 with rapamycin, or mTORC2 with the JR-AB2-011 compound. mTORC1-mediated phosphorylation of the downstream protein synthesis initiation factor 4E-Binding Protein 1 (4E-BP1) could result in the translation of proteins such as P-gp, see Supplementary Figure S1 (Wang et al., 2013), while mTORC2 has been implicated in the transcriptional regulation of this transporter (Kuo et al., 2002). While our results point towards a possible role of mTORC1 in regulating the protein expression of these transporters, we cannot definitively conclude that this subunit is majorly involved in the regulation of the transporters compared to mTORC2. Furthermore, we cannot rule out the involvement of mTORC2 in regulating the expression of these transporters following infection, since we did not assess their gene expression. To the best of our knowledge, this is the first study demonstrating that HIV-mediated activation of mTOR results in the induction of ABC drug efflux transporters. Overall, our results shed light on the mTOR pathway in regulating the expression of these transporters following exposure to pHIV_NL4-3_, however more work is needed with a larger sample size to further dissect the role of the mTOR subunits in this context.

We also demonstrated a role of mTOR in regulating the production of several proinflammatory cytokines following exposure to pHIV_NL4-3_. In agreement with our data, previous work also showed that inhibition of mTOR prevented T-cell activation and significantly decreased the secretion of several proinflammatory cytokines, including IL-6, TNFα and INFγ, in isolated T-cells (Herrero-Sánchez et al., 2016). Activation of mTORC2 potentially leads to downstream activation of NFκB (Kuo et al., 2002), a transcription factor known to promote the production of proinflammatory cytokines (Ronaldson and Bendayan, 2006; Ashraf et al., 2014b). Moreover, Wei et al. demonstrated that siRNA gene silencing and/or pharmacologic inhibition of the mTORC2 signaling pathway resulted in inhibition of LPS-induced NFκB phosphorylation and activation, and subsequently the production of proinflammatory cytokines IL-12, IL-23, INFγ and IL-17 in dendritic cells or T-cells cocultured with dendritic cells (Wei et al., 2015).

In classical secretory pathways, cytokines are loaded into membrane-bound vesicles, granules, or both, and delivered to the cell surface for release. However, various non-classical mechanisms are proposed for cytokines crossing the plasma membrane, including the use of membrane transporters, microvesicle shedding, or cell lysis (Nickel, 2003; Lacy and Stow, 2011). ABC drug efflux transporters have been implicated in the export of proinflammatory cytokines following activation of immune cells, but it remains controversial as to whether these transporters are involved in a non-classical membrane trafficking process as suggested by some groups (Drach et al., 1996; Zhang et al., 2006), or whether they mediate the secretion of other relevant physiological substrates, such as bioactive lipids, which in turn result in cytokine secretion as a secondary effect (Huang et al., 1999; Raggers et al., 2001). We detected significant and robust increases in proinflammatory cytokines IL6, TNFα and INFγ concentrations following exposure to pHIV_NL4-3_. However, inhibiting the function of P-gp and MRP1 significantly decreased the concentrations of these cytokines, demonstrating the potential roles of these transporters in cytokine export. These results are consistent with previous studies demonstrating that P-gp and MRP1 are involved in the transport of these cytokines in activated PBMCs or purified CD4^+^ T-cells (Drach et al., 1996; Frank et al., 2001; Pawlik et al., 2005; Zhang et al., 2006). At the mRNA level, inhibition of P-gp did not affect cytokine expression, suggesting that this transporter is only involved in the export of these cytokines. On the other hand, the MRP1 inhibitor MK571 attenuated the mRNA expression of these cytokines. It was previously proposed that MRP1 inhibition with MK571 led to higher intracellular accumulation of an eicosanoid substrate which, in turn, activated the transcriptional repressor peroxisome proliferator-activated receptor-γ (PPARγ), resulting in decreased cytokine production (Zhang et al., 2006). However, future studies are needed to investigate the effect of MK571 on PPARγ mediated cytokine reduction following HIV infection. In addition, at high concentrations MK571 could inhibit the function of MRP2 and MRP4, transporters that are also implicated in the export of various inflammatory mediators (van de Ven et al., 2009). Overall, these data suggest that MRP inhibition may indirectly reduce the production of proinflammatory cytokines, as well their export. While further work is needed, the current findings suggest an indirect role of ABC transporters in sustaining persistent HIV infection by contributing to proinflammatory cytokine release. The extent at which these transporters participate in inflammatory processes in people living with HIV necessitates further investigation.

A limitation of this study is that exposure to pHIV_NL4-3_ does not fully reflect HIV infection *in vivo*. The virus lacks viral enzymes reverse transcriptase and protease which are necessary to continue its replication cycle. We anticipate that the inductive effects of HIV on the transporters is most likely due to the viral proteins such as Tat and gp120. Hayashi et al. previously demonstrated that treatment of mouse brain microvascular endothelial cells with Tat induced the functional expression of P-gp through NF-κB mediated mechanisms (Hayashi et al., 2005). Furthermore, Tat induced MRP1 expression and function in mouse brain microvascular endothelial cells and astrocytes following activation of the mitogen-activated protein kinase signaling cascade (Hayashi et al., 2006). In addition, an induction of the expression and function of BCRP was reported in Tat-expressing Jurkat T-cell lines compared to control cells (Zhou et al., 2016). While the pHIV_NL4-3_ virus was pseudotyped with VSVG, we also demonstrated a potential role of gp120 in inducing T-cell activation and the expression of the transporters.

Overall, our results suggest that pHIV_NL4-3_ infection enhanced T-cell activation, and increased the expression of ABC drug efflux transporters potentially through the mTOR signaling pathway. Our study sheds light on molecular pathways that could contribute to persistent HIV infection, but further work is needed to elucidate the mTOR downstream signaling processes governing the upregulation of these ABC transporters, for example protein synthesis. HIV-induced activation of T-cells could result in viral replication and inflammation (Prasad et al., 2019). Furthermore, ABC drug efflux transporters are induced following HIV infection, and could restrict ARV penetration in T-cell reservoirs (Janneh et al., 2005, 2007; Fletcher et al., 2014; Whyte-Allman et al., 2020), as well as potentially contribute to HIV-associated inflammation indirectly through the secretion of proinflammatory cytokines (Drach et al., 1996). Therefore, the involvement of these transporters must be considered when developing therapeutic strategies to control HIV-associated immune activation and inflammation, as well as for improving the efficacy of ARVs in viral reservoirs and sanctuary sites.

## Data Availability

The original contributions presented in the study are included in the article/Supplementary Material, further inquiries can be directed to the corresponding author.
