# Supplementary material for: Regulation of ABC Drug Efflux Transporters in Human T-Cells Exposed to an HIV Pseudotype
Source: Front Pharmacol. 2021 Aug 4;12:711999. doi: 10.3389/fphar.2021.711999 (PMC8371480; doi:10.3389/fphar.2021.711999)
Supplement: Supplementary file 1 [file DataSheet2.PDF]

Supplemental Table S-1

Category	Marker/Antibodies	Fluorochrome or channel of detection	Company/ Cat #
Viability	Live/dead	Zombie aqua	Biolegend/ 423101
T-cells	Anti-CD3	PE-Cy7	Biolegend/ 317334
	Anti-CD4	BV-421	Biolegend/ 344631
	Anti-CD8	BV-605	Biolegend/ 344741
	Anti-CD69	PE/Dazzle 594	Biolegend/ 310941
Drug transporters	Anti-P-gp	PerCP-Cy5.5	Biolegend/ 348611
	Anti-BCRP	PE	Biolegend/ 332007
	Anti-MRP1	Alexa Fluor 647	Biolegend/ 370103
pmTOR	Phospho-mTOR (Ser2448)	PE	Ebioscience/12-9718-42

Table S-1. List of antibodies and fluorophores used in multicolor staining for flow cytometry.
