# Supplementary material for: Regulation of ABC Drug Efflux Transporters in Human T-Cells Exposed to an HIV Pseudotype
Source: Front Pharmacol. 2021 Aug 4;12:711999. doi: 10.3389/fphar.2021.711999 (PMC8371480; doi:10.3389/fphar.2021.711999)
Supplement: Supplementary file 2 [file DataSheet1.pdf]

Supplemental Figures

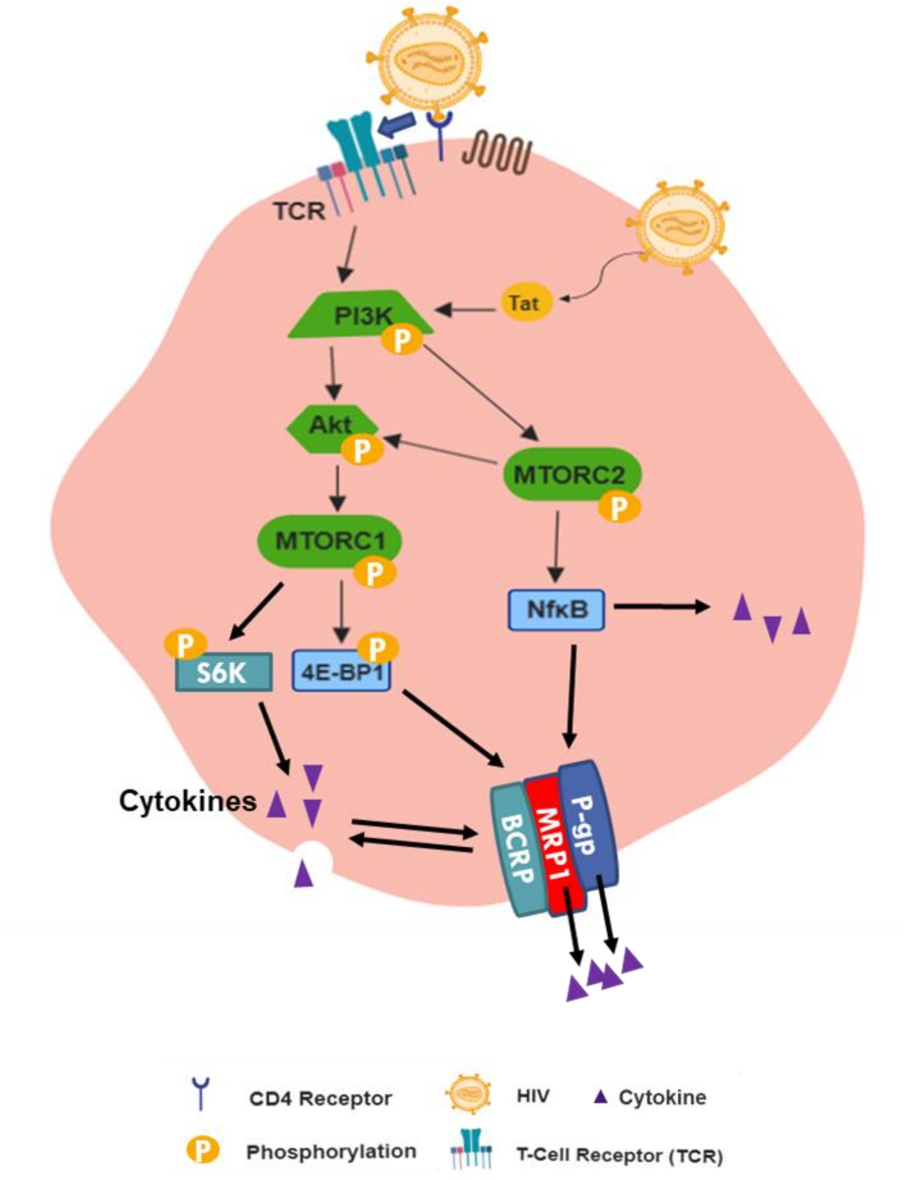

Supplemental Figure S-1. Potential involvement of mTOR in the regulation of drug efflux transporters and inflammatory response in HIV-infected T-cells. HIV envelope glycoprotein gp120 interacts with the CD4 receptor on T-cells which enhances the activation of the T-cell receptor. T-cell receptor activation results in downstream activation of the phosphatidylinositol 3-kinase (PI3K) upstream of mTOR. In addition, HIV Trans-Activator of Transcription protein (Tat) could directly induce activation of PI3K. PI3K activation leads to MTORC1 activation through phosphorylation of Akt, or direct activation of MTORC2. MTORC2 activation then leads to the prolonged activation of Akt and its substrates. mTOR activation leads to full T-cell activation and cytokine production. At the transcriptional level it is proposed that MTORC2 activation could regulate the expression of transporter genes such as MDR1, the gene which encodes P-gp in humans, potentially through an NfκB mediated mechanism. Additionally, MTORC1-mediated phosphorylation of 4E-Binding Protein 1 (4E-BP1) releases eukaryotic translation initiation factor 4E (eIF4E), which can then initiate translation of proteins including P-gp. As ABC transporters are implicated in cytokine release, they could potentially function in the secretion of cytokines as a primary export mechanism, or indirectly by influencing classical secretory pathways.

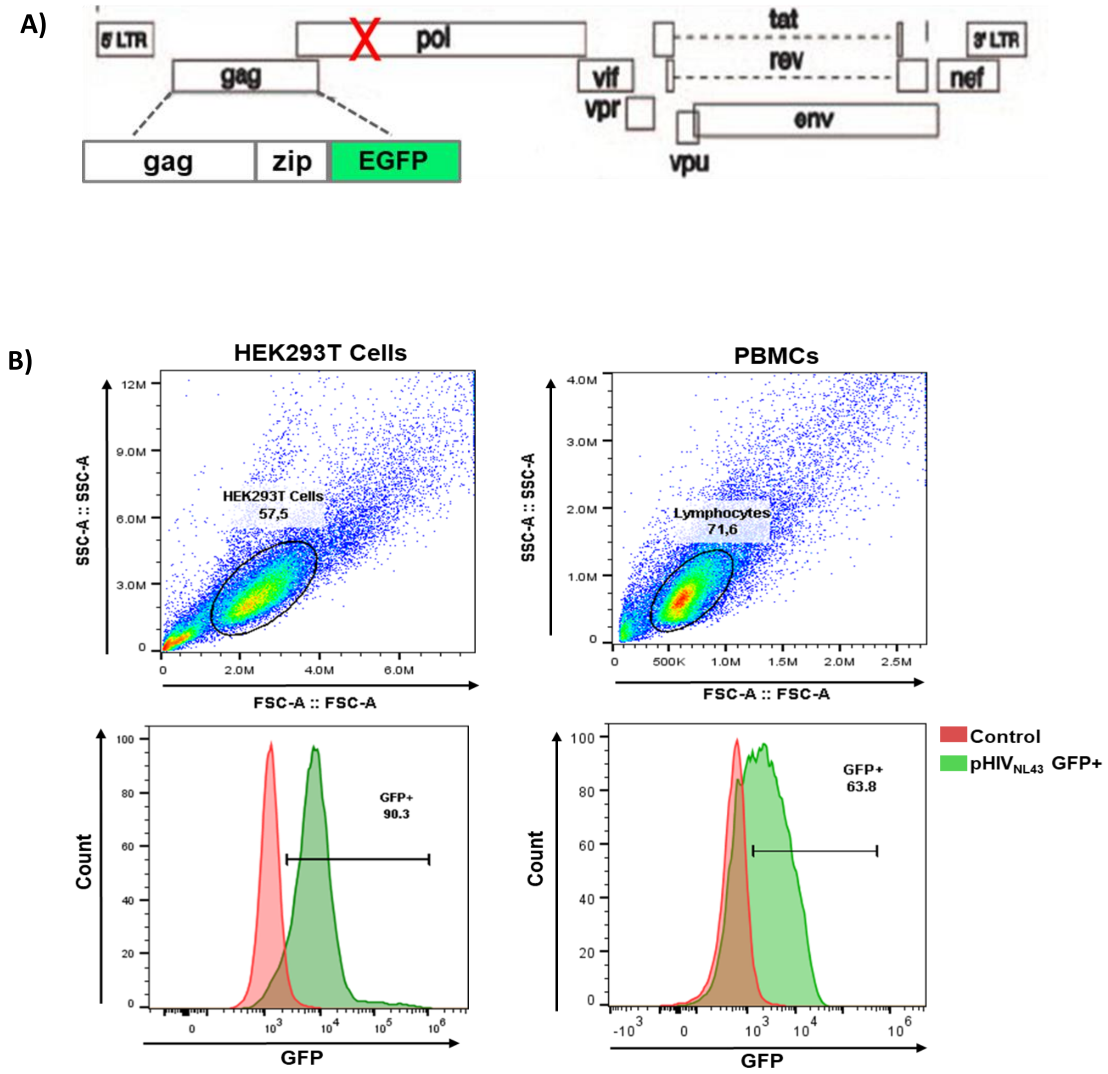

Supplemental Figure S-2. pHIV$_{NL4-3}$ virus infectivity. (A) Schematic of HIV$_{NL4-3}$ provirus (pHIV$_{NL4-3}$) demonstrating the nine viral genes and 2 LTR, with deletion in regions of pol gene and modifications of the gag gene to express enhanced GFP (EGFP). (B) Dot plots demonstrating viable HEK293T cells or peripheral blood lymphocytes gated using light scattering properties (top panels), and representative histograms demonstrating the frequency of HEK293T cells or peripheral blood lymphocytes carrying GFP-expressing pHIV$_{NL4-3}$ (GFP+) (bottom panels).

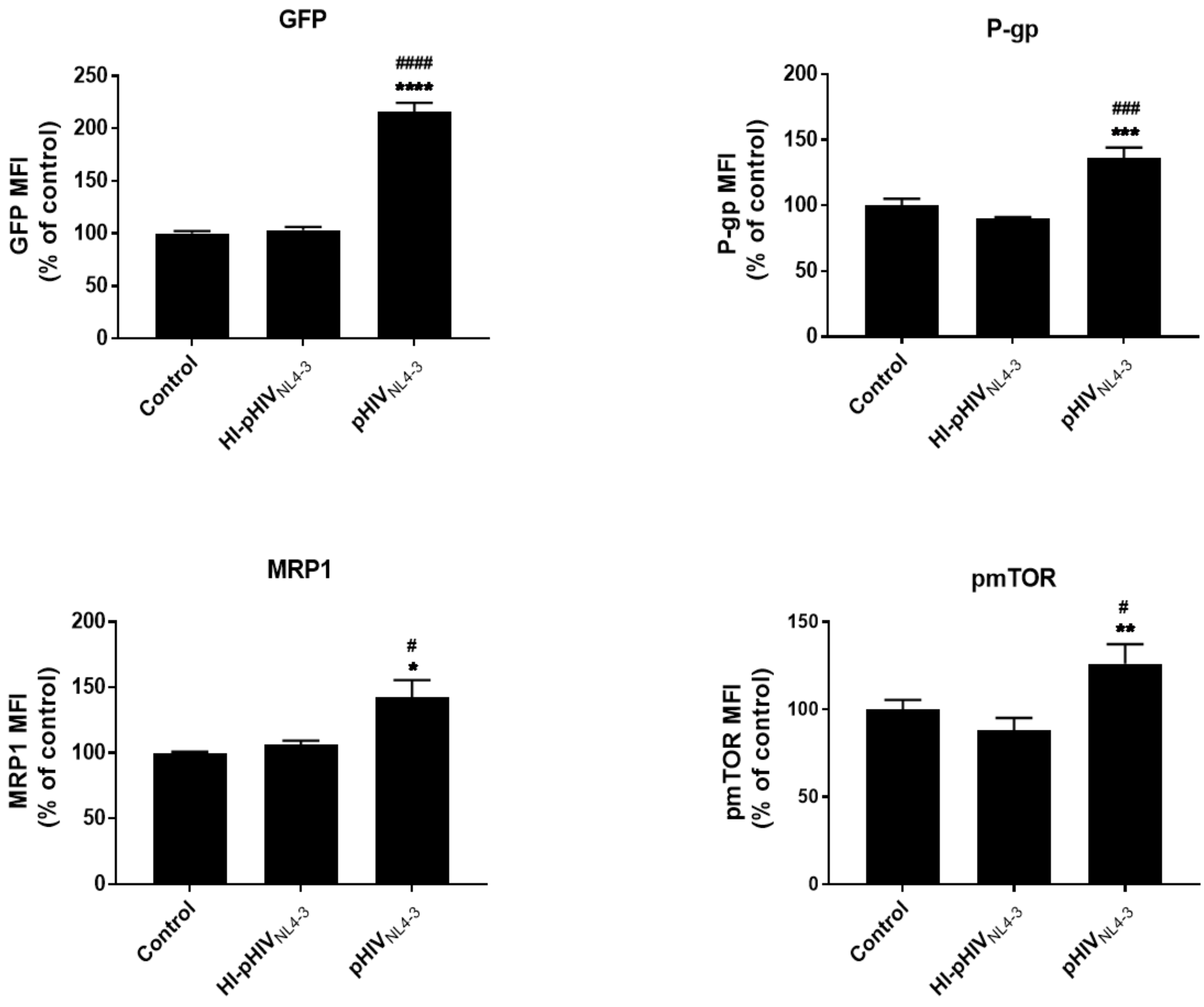

Supplemental Figure S-3. Activity of heat-inactivated pHIV$_{NL4-3}$ in CD4+ T-cells. Heat-inactivation was achieved by heating pHIV$_{NL4-3}$ at 57 °C for 45 mins (HI-pHIV$_{NL4-3}$). GFP expression was then examined in CD4+ T-cells exposed to the inactivated virus compared to live virus (pHIV$_{NL4-3}$) and negative control. The effect of HI-pHIV$_{NL4-3}$ on the expression of transporters and pmTOR was assessed. Results are shown as mean percent change ± S.E.M. in the expression (median fluorescence intensity, MFI) of GFP, P-gp, MRP1 and pmTOR. Statistical analyses were performed using one-way ANOVA with Bonferroni's multiple comparisons test. *, statistically significant difference compared to control; #, statistically significant difference compared to HI-pHIV$_{NL4-3}$, n=3 donors. *, $p<0.05$; **, $p<0.01$; ***, $p<0.001$; ****, $p<0.0001$. #, $p<0.05$; ###, $p<0.001$; ####, $p<0.0001$.
